# Anti-N-Methyl-D-Aspartate (NMDA) Receptor Encephalitis Secondary to an Ovarian Dermoid Cyst

**DOI:** 10.7759/cureus.67193

**Published:** 2024-08-19

**Authors:** Nicolette Casarcia, Sunni A Coyne, Hussain Rawiji

**Affiliations:** 1 Obstetrics and Gynecology, Lake Erie College of Osteopathic Medicine, Bradenton, USA; 2 Obstetrics and Gynecology, AdventHealth Florida, Orange City, USA

**Keywords:** encephalopathy, paraneoplastic manifestation, ovarian cyst, glucocorticoid therapy, dermoid cysts, anti-nmdar encephalitis

## Abstract

Dermoid cysts, or mature cystic teratomas, are germ cell neoplasms that can arise on the ovaries. Being of germ cell origin, such cysts can have extensive variance in presentation, including a rare paraneoplastic effect where they produce N-methyl-D-aspartate receptor (NMDAR) antibodies, resulting in anti-NMDAR encephalitis. This can cause various neuropsychiatric symptoms, including confusion, hallucinations, psychosis, disorientation, and a change in cognition. This case study presents the unusual occurrence of a 39-year-old female patient who presented to the emergency department with encephalitis, headaches, and auditory hallucinations after recent glucocorticoid use. Through an extensive workup, imaging, and various physician consults, the patient was diagnosed with anti-NMDAR encephalitis secondary to a paraneoplastic effect originating from an ovarian dermoid cyst.

## Introduction

Ovarian dermoid cysts, or mature cystic teratomas, are relatively common benign germ cell neoplasms that are typically filled with fluid and may contain various tissues, such as teeth, hair, or skin. These germ cell cysts are typically present at birth and may be located bilaterally. Patients with these cysts may have various presentations, including but not limited to pain, ovarian torsion, or cystic rupture [1]. However, most ovarian dermoid cysts do not cause symptoms and are discovered incidentally on imaging [2]. Rarely, patients may experience nausea, dyspareunia, or a poor appetite. Dermoid cysts account for approximately 15% of ovarian cysts and are more common in females who are 20 to 40 years of age. Although typically benign, these slow-growing neoplasms may become cancerous and a cause for concern if they cause pain, cystic rupture, ovarian torsion, or, rarely, psychiatric symptoms [3].

Anti-N-methyl-D-aspartate receptor (NMDAR) encephalitis is an autoimmune or paraneoplastic condition in which IgG antibodies are produced to this receptor. Such antibodies typically target the NR1 subunit of this receptor. Cerebrospinal fluid analysis can identify the presence of these antibodies, which aids in the diagnosis. This results in numerous neuropsychiatric effects, such as, but not limited to, confusion, disorientation, psychosis, or paranoia. Prompt identification and treatment of this disease is crucial for patient well-being [4]. This paraneoplastic effect can arise from many different forms of malignant and benign neoplasms, such as small-cell lung carcinomas, uterine adenocarcinoma, prostate adenocarcinoma, Hodgkin lymphoma, pineal dysgerminomas, neuroblastoma, and pancreatic neuroendocrine tumors, in addition to ovarian dermoid cysts [5]. Here, we introduce a case that features a 39-year-old female who presented with an acute onset of worsening neuropsychiatric symptoms from NMDAR antibodies produced by an ovarian dermoid cyst.

## Case presentation

A 39-year-old female with a past medical history of chronic ankle pain was brought to the emergency department due to a headache and auditory hallucinations for three days. Her family stated that they were concerned with her decline in function, which included confusion, disorientation, hallucinations, and an inability to complete her everyday tasks. She reported chronic leg and ankle pain, which she had been treating with over-the-counter medications for approximately two years. Due to worsening pain, she obtained an intra-articular steroid injection four days prior and oral steroids three days prior to presenting to the hospital. Her symptoms began shortly after starting these medications. The initial exam found her to be afebrile with adequate oxygenation but mild tachycardia at 116 beats per minute (bpm) and a blood pressure of 131/71 mmHg. Her physical exam was unremarkable at that time, with appropriate breath sounds and mild tachycardia but a regular cardiac rhythm. Additionally, she was alert and oriented to person, place, and time. She was started on intravenous (IV) fluids and received supportive care. The oral steroid was discontinued, and the patient was admitted for observation with a psychiatric consult.

Upon further questioning, the patient admitted to new-onset forgetfulness, difficulty concentrating, visual and auditory hallucinations, and paranoia. She stated that the hallucinations were worse at night, leading to difficulty sleeping. She had appropriate thought content and judgment with no history of mood symptoms, suicidal or homicidal thoughts, mania, or psychotic symptoms. At that point, the patient appeared fatigued, had minimal eye contact, and had a slow speech rate with an irregular rhythm. She portrayed an anxious mood and a flat affect. The patient was able to state her age and birthdate, but not the date she arrived at the hospital due to disorientation at that point in time.

Over the next few days, further laboratory workup yielded minimal results, including a negative drug screen, unremarkable blood tests, and a normal acetaminophen level. However, her urinalysis revealed 3+ protein with trace bacteria and mucus. Furthermore, her imaging was unremarkable, including a chest X-ray, head CT and MRI without IV contrast, a standard lumbar puncture, a repeat head MRI, and a CT scan of the chest, abdomen, and pelvis. An abnormal electroencephalogram (EEG) reported intermittent generalized theta activity, which was suggestive of mild, nonspecific, generalized cerebral dysfunction. There were no epileptiform discharges or electrographic seizures noted. A transabdominal pelvic ultrasound resulted in a limited evaluation of the pelvis, no visualization of the left ovary, and an unremarkable right ovary. Throughout the patient's hospital stay, her condition continued to worsen with major cognitive changes, including agitation, disorientation, paranoia, and confusion, despite intervention. An infectious disease physician had suggested anti-NMDAR encephalitis, but the workup and imaging did not initially support this diagnosis. Despite several consults, the source of her condition could not be confidently identified.

Upon an autoimmune workup, the patient had a 1:80 titer of NMDAR antibodies (ref range: <1:1) in her cerebrospinal fluid (CSF). CSF analysis also showed elevated white blood cells with a lymphocytic predominance, although the remaining CSF autoimmune titers were unremarkable. Her serum voltage-gated potassium channel antibody was elevated at 56 pmol/L (ref range: <31 pmol/L), which can occur in paraneoplastic neurological and encephalopathic syndromes [6]. Due to the patient's positive NMDAR antibody titer, it was decided that a paraneoplastic disorder needed to be ruled out, particularly an occult ovarian dermoid cyst in this young female patient.

**Figure 1:**
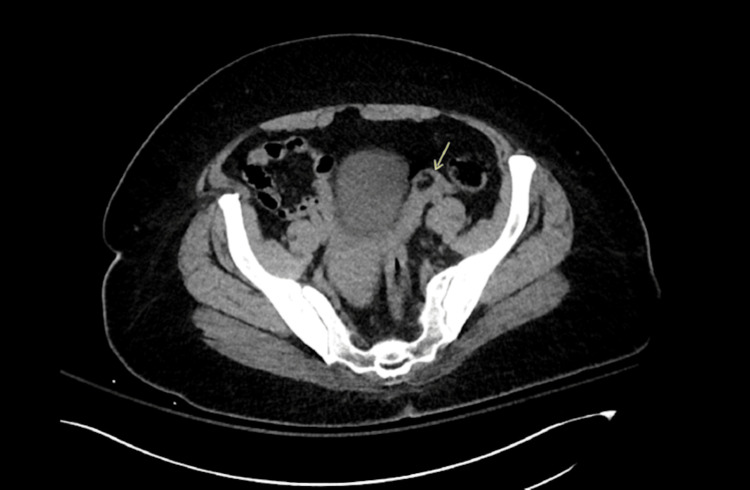
A CT scan of the pelvis revealing a 1.6 cm left ovarian dermoid cyst.

Due to this, a repeat pelvic ultrasound was completed but showed no visualization of the bilateral ovaries. In addition, a transvaginal ultrasound could not be consented to due to the patient's state at that time. Further, an MRI of the patient's pelvis without IV contrast was markedly incomplete due to the patient's confusion and refusal of the scan. However, upon the radiologist's further review of past imaging, it was noted that the previous CT scan of the chest, abdomen, and pelvis demonstrated a 1.6 cm dermoid cyst on the left ovary (Figure 1).

The patient was diagnosed with anti-NMDAR encephalitis secondary to a left ovarian dermoid cyst. She was prescribed 1,000 mg of IV rituximab and 100 mg of Seroquel as needed and scheduled for an exploratory laparotomy with left salpingectomy. The cyst was removed, and the pathology report confirmed a mature dermoid cyst. A few days following the surgery, she had a gradual reduction of her symptoms, including significantly decreased confusion, combativeness, and cessation of her anxiety and restlessness. She was discharged and instructed to follow up with a gynecology/oncology specialist for observation and further consideration of an exploratory laparotomy, as only one ovarian dermoid cyst was seen at the time of surgery, but they can be bilateral or found in other parts of the body.

## Discussion

Ovarian dermoid cysts, or mature cystic teratomas, are the most common benign ovarian tumors in adult and adolescent women [7]. Teratomas are typically uncovered incidentally on imaging but may sometimes cause ovarian torsion or cystic rupture with excessive growth. Rarely, patients may present with neuropsychiatric symptoms, suggesting anti-NMDAR encephalitis, as in this patient [8].

Teratomas originate from germ cell layers and may contain mesoderm, ectoderm, and endoderm, which allow them to produce skin, hair, sweat glands, teeth, and numerous other mature tissues within their structure [1]. Interestingly, dermoid cysts may also be present in the testes and, less commonly, in the sacrococcygeal region, head, spine, mediastinum, or neck [1]. Ovarian dermoid cysts may be diagnosed with a transvaginal or abdominal ultrasound. If an ultrasound is inconclusive, an MRI may be utilized [9]. Depending on the overall state and mental status of the patient, obtaining these images may be difficult, as in this case. Ovarian dermoid cysts are most often benign and do not need to be surgically removed. However, surgery may be attempted if the cysts are large, approximately greater than 5 cm, cause symptoms, or have a high likelihood of becoming malignant. Commonly, cysts will not regress on their own and may only be removed through treatment if indicated. Further, in patients with anti-NMDAR encephalitis, removal of the tumor is essential for recovery [10]. Malignant transformation is most common in individuals between 40 and 60 years of age but typically occurs in those between 30 and 70 years. Approximately 1%-3% of benign neoplasms become malignant, which are more typical in cysts that grow rapidly or are greater than 10 cm in diameter. These malignancies are typically squamous cell carcinoma but may also be transitional cell carcinoma, malignant melanoma, choriocarcinoma, carcinoids, sarcomas, and adenocarcinoma [1]. Additionally, 10%-15% of dermoid cysts are bilateral. Surgical treatments include ovarian cystectomy or oophorectomy via laparoscopy or laparotomy [11].

Anti-NMDAR encephalitis is an autoimmune response of IgG antibodies directed against the NR1 subunit of the NMDA receptor, resulting in swelling and disruption of brain signaling [12]. This phenomenon can induce a multitude of behavioral changes, including paranoia, hallucinations, or aggression, and experiences in patients such as cognitive and memory deficits, speech or movement disorders, seizures, headaches, autonomic dysfunction, or even loss of consciousness [13]. Typically, patients are first referred for psychiatric workups due to the presentation and onset of symptoms. Due to this, there is often a delay in diagnosis, which commonly impacts patients' clinical courses, resulting in the worsening of symptoms prior to diagnosis. Although this condition may be due to a paraneoplastic effect, 40% of cases do not have a concurrent neoplasm. In those with a coinciding neoplasm, small-cell lung cancer, thymoma, breast cancer, ovarian cancer, and testicular cancer tend to be the most common [14]. Interestingly, benign ovarian dermoid cysts have been correlated with anti-NMDAR encephalitis, as seen in this patient. These cases have only recently been documented, and since 2007, the number of case reports has progressively increased, suggesting that ovarian teratomas may be a more common cause of anti-NMDAR encephalitis than originally proposed [15].

Anti-NMDAR encephalitis can be diagnosed by clinical features, EEG, and CSF analysis and confirmed with CSF antibody detection (Table 1). The first-line treatment of this disorder includes the removal of a tumor, if present, and immunomodulatory therapy, such as rituximab [16]. Without intervention, this condition can be fatal, and a prompt diagnosis results in a more favorable prognosis [17].

**Table 1:** Diagnostic criteria for anti-NMDAR encephalitis. *Antibody testing should include testing of CSF. If only serum is available, confirmatory tests should be included in addition to cell-based assays. **In the presence of a systemic teratoma, a diagnosis can be made in the presence of three groups of symptoms [18]. NMDAR: N-methyl-D-aspartate receptor; CSF: cerebrospinal fluid.

Likelihood of Anti-NMDAR Encephalitis	Findings
Definite diagnosis	IgG anti-GluN1 antibodies* in the presence of one or more of the six major groups of symptoms, after reasonable exclusion of other disorders
Probable diagnosis	Rapid onset (<3 months) of at least four of the following major groups of symptoms**: abnormal behavior or cognitive dysfunction, speech dysfunction, autonomic dysfunction or central hypoventilation, decreased level of consciousness, movement disorder, dyskinesias, or rigidity/abnormal posture, seizures
	At least one of the following laboratory results: abnormal EEG (focal or diffuse slow or disorganized activity, epileptic activity, or extreme delta brush), CSF with pleocytosis or oligoclonal bands
	Reasonable exclusion of other disorders

In this particular case, the patient's symptoms presented acutely after oral and intra-articular steroid use. While no definitive cause of this occurrence was discovered, corticosteroids typically inhibit inflammatory responses and are commonly used to treat paraneoplastic syndromes [19]. It is not completely understood why steroids would unmask this immune-mediated reaction in this patient, although it is unlikely that this was the result of a steroid-induced psychosis occurring with subsyndromal anti-NMDAR encephalitis, as the steroids were discontinued and the patient continued to decline over the next few days to weeks. In addition, it was only after the removal of the ovarian cyst that the patient had clinical improvement.

## Conclusions

Although rare, it is important to consider ovarian dermoid cysts when a patient presents with encephalopathy and anti-NMDAR encephalitis. This condition may be detrimental to one's health and cognitive function if not identified early and treated promptly. While ovarian teratomas typically remain benign and rarely cause substantial symptoms in most patients, healthcare professionals must understand the effects that may occur when they are present. In addition, this case signifies the importance of understanding the patient's history and obtaining information from family members, as this may greatly aid in the diagnosis and understanding of the patient's baseline status and current disease process, especially when they are acutely encephalopathic.

In the case presented, prompt diagnosis and treatment were utterly important to shorten the duration of symptoms this patient experienced. This 39-year-old female suffered from hallucinations, paranoia, and worsening encephalopathy throughout her hospital stay. Only after the identification and resection of the ovarian dermoid cyst did she begin to improve. Healthcare providers must consider ovarian dermoid cysts in the case of psychosis, as rapid identification and treatment can ensure safety and improve the lives of affected patients.

## References

[1] Ahmed A, Lotfollahzadeh S (2024). Cystic teratoma. https://www.statpearls.com/point-of-care/29963.

[2] Saleh M, Bhosale P, Menias CO, Ramalingam P, Jensen C, Iyer R, Ganeshan D (2021). Ovarian teratomas: clinical features, imaging findings and management. Abdom Radiol.

[3] (2024). Teratoma. https://my.clevelandclinic.org/health/diseases/22074-teratoma.

[4] (2024). Anti-NMDAR encephalitis. https://www.statpearls.com/point-of-care/17673#:~:text=This%20autoimmune%20encephalitis%20develops%20due,the%20receptor%2Ddependent%20synaptic%20currents..

[5] Bost C, Chanson E, Picard G (2018). Malignant tumors in autoimmune encephalitis with anti-NMDA receptor antibodies. J Neurol.

[6] Jammoul A, Shayya L, Mente K, Li J, Rae-Grant A, Li Y (2016). Clinical utility of seropositive voltage-gated potassium channel-complex antibody. Neurol Clin Pract.

[7] St Louis M, Mangal R, Stead TS, Sosa M, Ganti L (2022). Ovarian dermoid tumor. Cureus.

[8] Cong L, Wang S, Yeung SY, Lee JH, Chung JP, Chan DY (2023). Mature cystic teratoma: an integrated review. Int J Mol Sci.

[9] Sahin H, Abdullazade S, Sanci M (2017). Mature cystic teratoma of the ovary: a cutting edge overview on imaging features. Insights Imaging.

[10] Dalmau J, Rosenfeld MR (2008). Paraneoplastic syndromes of the CNS. Lancet Neurol.

[11] Cleveland Clinic (2024). Ovarian dermoid cyst. https://my.clevelandclinic.org/health/diseases/23931-ovarian-dermoid-cyst.

[12] Kayser MS, Titulaer MJ, Gresa-Arribas N, Dalmau J (2013). Frequency and characteristics of isolated psychiatric episodes in anti-N-methyl-D-aspartate receptor encephalitis. JAMA Neurol.

[13] Perelman School of Medicine (2024). Anti-NMDAR encephalitis. https://www.med.upenn.edu/autoimmuneneurology/nmdar-encephalitis.html.

[14] Punja M, Pomerleau AC, Devlin JJ, Morgan BW, Schier JG, Schwartz MD (2013). Anti-N-methyl-D-aspartate receptor (anti-NMDAR) encephalitis: an etiology worth considering in the differential diagnosis of delirium. Clin Toxicol.

[15] Acién P, Acién M, Ruiz-Maciá E, Martín-Estefanía C (2014). Ovarian teratoma-associated anti-NMDAR encephalitis: a systematic review of reported cases. Orphanet J Rare Dis.

[16] Dalmau J, Rosenfeld M (2024). Autoimmune (including paraneoplastic) encephalitis: clinical features and diagnosis. UpToDate.

[17] Mitra AD, Afify A (2018). Ovarian teratoma associated anti-N-methyl-D-aspartate receptor encephalitis: a difficult diagnosis with a favorable prognosis. Autops Case Rep.

[18] Graus F, Titulaer MJ, Balu R (2016). A clinical approach to diagnosis of autoimmune encephalitis. Lancet Neurol.

[19] (2024). Paraneoplastic syndromes. https://my.clevelandclinic.org/health/diseases/17938-paraneoplastic-syndromes.

